# Y-Chromosome Variation in Altaian Kazakhs Reveals a Common Paternal Gene Pool for Kazakhs and the Influence of Mongolian Expansions

**DOI:** 10.1371/journal.pone.0017548

**Published:** 2011-03-11

**Authors:** Matthew C. Dulik, Ludmila P. Osipova, Theodore G. Schurr

**Affiliations:** 1 Department of Anthropology, University of Pennsylvania, Philadelphia, Pennsylvania, United States of America; 2 Institute of Cytology and Genetics, Siberian Branch of the Russian Academy of Sciences (SB RAS), Novosibirsk, Russia; Erasmus University Medical Center, Netherlands

## Abstract

Kazakh populations have traditionally lived as nomadic pastoralists that seasonally migrate across the steppe and surrounding mountain ranges in Kazakhstan and southern Siberia. To clarify their population history from a paternal perspective, we analyzed the non-recombining portion of the Y-chromosome from Kazakh populations living in southern Altai Republic, Russia, using a high-resolution analysis of 60 biallelic markers and 17 STRs. We noted distinct differences in the patterns of genetic variation between maternal and paternal genetic systems in the Altaian Kazakhs. While they possess a variety of East and West Eurasian mtDNA haplogroups, only three East Eurasian paternal haplogroups appear at significant frequencies (C3*, C3c and O3a3c*). In addition, the Y-STR data revealed low genetic diversity within these lineages. Analysis of the combined biallelic and STR data also demonstrated genetic differences among Kazakh populations from across Central Asia. The observed differences between Altaian Kazakhs and indigenous Kazakhs were not the result of admixture between Altaian Kazakhs and indigenous Altaians. Overall, the shared paternal ancestry of Kazakhs differentiates them from other Central Asian populations. In addition, all of them showed evidence of genetic influence by the 13^th^ century CE Mongol Empire. Ultimately, the social and cultural traditions of the Kazakhs shaped their current pattern of genetic variation.

## Introduction

The Kazakhs first emerged as a political unit during the 15^th^ century CE in the region that is now southern Kazakhstan. After the Uzbek Khanate lost authority over the region north of Syr Darya due to Oirat incursions, remnants of the old Mongolian White Horde gained control over the area, forming a new political entity, the Kazakh Khanate [1]. This political group contained a mixture of peoples, having incorporated Uzbek defectors, indigenous peoples of the region and immigrants from Dasht-i-Qipchak [1], [2]. During the 16^th^ century, the Kazakhs divided to form three *Zhüz*, called the Great, Middle and Small Hordes [3]. These *Zhüz* were not constructed along lines of common descent, but rather reflected the political divisions of the population and the unique geography of the region [1].

Coming under increased pressure from the Kalmyks in the 18^th^ century, independent Kazakh rule ended, with Russia taking control of Kazakh lands in the mid-1700s. By this time, a distinctive Kazakh ethnic group had formed, resulting in a shared common history, language and culture among the three Kazakh *Zhüz*. It was also at this time that some Kazakhs moved to the steppe lands northeast of Lake Zaysan in Kazakhstan. During the 19^th^ and into the 20^th^ centuries, Kazakhs migrated through Xinjiang, China, and eventually spread north around the Altai Mountains in western Mongolia and southern Russia. Sources suggest that these Kazakhs came from the Middle *Zhüz*, although multiple eastward migrations likely occurred [4], [5].

Kazakh culture derived from the nomadic cultures that were dominant among Turkic tribes living on the Central Asian steppe [4]. In many ways, their culture resembles the economies of historically known groups that previously resided in the same region (i.e., Scythians, Turks, Mongols) [3]. It relies heavily upon a pastoral economy, where prestige is gained by the size of one's herd [1]. The persistent need to sustain their herds also requires a semi-nomadic lifestyle with migrations between summer and winter locations [6]. Moreover, this pastoralist existence is central to their cultural identity [1], [4].

Despite these deeper connections to numerous Turkic-speaking populations, the Mongol Empire strongly influenced Kazakh political and social structure. This influence was of such significance that the Kazakh aristocracy legitimized its authority by claiming direct ancestry from Genghis Khan (whether such connections were imaginary or real) through the *süök* system and largely supported through extensive genealogies [1], [4]. Following the traditions arising in Turkic and Mongolic tribes from which the Uzbeks and Kazakhs emerged, their society was a patrilineal tribal system in which descent groups formed around closely related men. Historically, only the wealthiest Kazakhs practiced polygyny, although such practices do not occur today [4].

Policies enforced by the Russian government also had a significant impact on the lives of the Central Asian steppe nomads. Historical and ethnographic materials show that, through Russian acculturation, Kazakhs took up a semi-nomadic economy, which relied on their migratory *auls* becoming sedentary, with only some Kazakhs maintaining seasonal migrations [4]. Through this process, villages became more reliant on agricultural products, and often the poorer of the Kazakh families had no choice but to adopt these new subsistence practices. The clan and *süök* social structures that helped to guide marriage practices lost importance, although patrilineal customs prevailed. Thus, the *auls* still consist mostly of extended families that can be recognized as closely related descent groups, and maintain some semblance of their previous culture practices.

Previous efforts to understand genetic variation within Altaian Kazakhs revealed a unique pattern of mtDNA diversity which differed from that of indigenous Kazakhs (i.e., those living in Kazakhstan proper) [7]. This pattern likely reflects the Altaian Kazakhs' eastward migration(s) from their original homeland. Accordingly, our mtDNA analysis showed that Altaian Kazakh populations were extremely diverse, having high levels of haplotype diversity (h = 0.997±0.001). Their mtDNAs belonged to roughly 66% East Eurasian and 33% West Eurasian haplogroups [7]. This frequency of West Eurasian haplogroups was higher than those seen in neighboring populations of Kazakh, Kyrgyz and Uyghur populations [8]. In addition, while Altaian Kazakh villages showed some degree of genetic differentiation, they appeared to share a common biological ancestry, suggesting that the observed differences were attributable to the presence of clan structure or closely related descent groups. Overall, the mtDNA genetic diversity in Altaian Kazakh populations suggested a rich, complex population history.

It is within this framework that we investigated the paternal genetic history of Altaian Kazakhs by characterizing the non-recombining Y-chromosome (NRY) variation through analysis of high-resolution biallelic markers and short tandem repeat (STR) typing. This approach allowed us to investigate several aspects of the history of this population. To begin with, we assessed the genetic relationship between Altaian Kazakhs and indigenous Kazakhs to better understand the origins and differentiation of the Kazakh ethnic group. We also examined the extent of historical admixture between Altaian Kazakhs and their indigenous Altaian neighbors in the genetically diverse Altai-Sayan region of Siberia. At a broader scale, we explored the relationships between Kazakh and Central Asian populations in an effort to clarify the history of Turkic-speaking groups. We further examined the possible genetic influence of Mongol expansions (Mongol Empire) on the peoples who later formed the Kazakhs, as well as their impact on Turkic-speaking populations across Central Asia. Our results indicate that Kazakhs have low levels of paternal genetic diversity, and share a common paternal ancestry that has been influenced by Genghis Khan's descendants. Kazakh culture has also played a central role in shaping this genetic variation through constraints on population size and marriage practices within traditional Kazakh social structure.

## Results

### Kazakh Haplogroup Diversity

Paternal genetic variation within Altaian Kazakhs was rather low. Some 85% of the Altaian Kazakh Y-chromosomes belonged to one of only three haplogroups (**Table 1**). RPS4Y-derived haplogroups predominated, and accounted for nearly 60% of the sample set, with C3* and C3c comprising this group of Y-chromosomes (20.2% and 39.5%, respectively). O3a3c* was the third common haplogroup, and encompassed 26.1% of the total male population. Also present were haplogroups J2a, G1, G2a, Q1a3*, R1a1a*, R1b1b1 and T, although each of these accounted for less than 5% of the entire sample set.

**Table 1:** High-resolution haplogroup classification for Altaian Kazakhs (by location).

Haplogroup	SW Altai	SE Altai	Total
C3*	10 (0.333)	14 (0.157)	24 (0.202)
C3c	7 (0.233)	40 (0.449)	47 (0.395)
G1	3 (0.100)	1 (0.011)	4 (0.034)
G2a	2 (0.067)		2 (0.017)
J2a	1 (0.033)	4 (0.045)	5 (0.042)
O3a3c*	1 (0.033)	30 (0.337)	31 (0.261)
Q1a3*	1 (0.033)		1 (0.008)
R1a1* (xR1a1a-e)	1 (0.033)		1 (0.008)
R1b1b1	3 (0.100)		3 (0.025)
T	1 (0.033)		1 (0.008)
**Grand Total**	**30**	**89**	**119**

**Figure 1:**
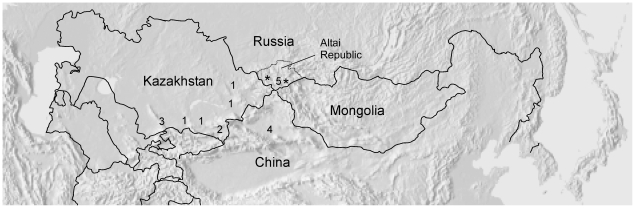
Kazakh populations analyzed in this study. The asterisks (*) denote the locations of Altaian Kazakh populations sampled for this study. The locations of comparative Kazakh populations are shown with each corresponding number: Altaian Kazakh [this study], Kazakh1 [10], [13], Kazakh2 [12], Kazakh3 [11], Kazakh4 [40], and Kazakh5 [41]. Kazakh1 represents samples that were collected from four locations [10], [13].

The Altaian Kazakh samples came from two areas of the southern Altai Republic, the southwestern (SW) and southeastern (SE) regions (**Figure 1**). Both locations had C3*, C3c, O3a3c*, J2a and G1 haplotypes. The SW Altaian Kazakhs had several additional haplogroups not found in the larger SE population, with three individuals having R1b1b1, two having G2a and one individual each having Q1a3*, R1a1a* and T. The more diverse set of haplogroups in the SW Altaian Kazakhs may point to a different population history for this location or perhaps its relative isolation from the greater Kazakh population. The mtDNA data also supported this interpretation, and suggested possible admixture between Kazakh and Russian residents in this area [7].

However, the NRY results showed no recognizable admixture between Kazakhs and Russians. The most frequent haplogroups present in southern Russia belong to R1a, N1c and I1b [9]. Although a single R1a1a* lineage appeared in Altaian Kazakhs, it was very similar to ones seen in the indigenous Altai-kizhi (unpublished data). In addition, N1c and I1b were not found in any Kazakh populations. Thus, while the mtDNA data suggested admixture with Russians, the NRY data suggested limited admixture with indigenous populations.

Comparisons of paternal haplogroup frequencies with Kazakhs sampled from four locations in eastern Kazakhstan (indigenous Kazakh) [10] revealed a pattern consistent with that seen in the Altaian Kazakhs (**Table 2**). The majority of indigenous Kazakh Y-chromosomes belonged to C-derived haplogroups, which were at higher frequencies than seen in Altaian Kazakh populations (66.7% versus 59.5%). However, one significant difference was the much greater frequency of C3c in the Kazakhs from Kazakhstan. C3c comprised 39.5% of all samples in Altaian Kazakhs, whereas it made up 57.4% of the indigenous Kazakh samples. In addition, the two populations differed substantially in the frequency of haplogroup O. Some 26% of Altaian Kazakhs possessed the M122 marker, while it was present in only 9% of the indigenous Kazakhs. In this regard, O3a3c* is the second most frequent haplogroup among SE Altaian Kazakhs.

**Table 2:** Low-resolution haplogroup classification for Kazakh populations.

Haplogroup	Altaian Kazakh	Kazakh1
C (xC3c)	24 (0.20)	5 (0.09)
C3c	47 (0.39)	31 (0.57)
D		1 (0.02)
F (xJ)	6 (0.05)	1 (0.02)
J	5 (0.04)	
K (xN1c, O, P)	1 (0.01)	
N1c		1 (0.02)
O (xO1, O2a, O3)		1 (0.02)
O3	31 (0.26)	5 (0.09)
P (xR1)	4 (0.03)	4 (0.08)
R1	1 (0.01)	5 (0.09)
**Grand Total**	**119**	**54**
**Reference**	This study	[22]

*Haplogroups E, O1 and O2a are not shown in Table 2 because they are not present in Kazakh populations, although they are part of the 14-haplogroup profile used in the haplogroup analysis and PCA.

† SNP data was not available for Kazakh2 [11] and Kazakh3 [10].

Despite these similarities, Altaian Kazakhs and indigenous Kazakhs showed a fair amount of difference in terms of their NRY haplogroup composition. Altaian Kazakhs tended to have higher frequencies of G1, G2 and J2 haplotypes, while indigenous Kazakhs had higher frequencies of Q and R1a. Thus, while the general pattern of paternal genetic variation was similar in these two groups, suggesting that they shared a common paternal ancestry, there were also specific genetic differences between them that likely reflected their respective genetic histories.

### Altaian Kazakh Haplotype Diversity

**Figure 2:**
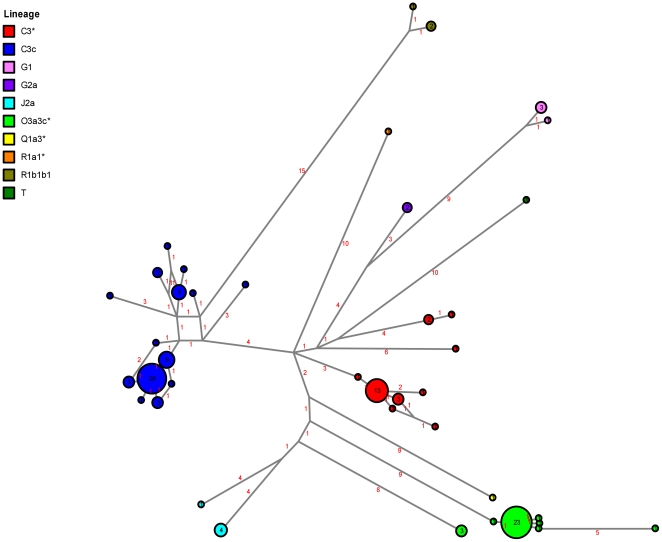
Reduced median-median joining network of Altaian Kazakhs using 14-STR haplotypes.

Analysis of 17 fast evolving Y-STRs provided additional details that helped to elucidate the paternal diversity among Kazakh populations. In total, we identified 51 haplotypes among the 119 Altaian Kazakhs (**Figure 2**; **Table S1**). There was a large amount of variation between the two sample locations for each of the three major haplogroups. Interestingly, only two haplotypes were shared between regions. One was more frequent among SW Altaian Kazakhs (haplotype #3), while the other appeared at low frequencies in both locations (haplotype #1). Both of these haplotypes belonged to haplogroup C3*. No other haplotypes were shared when considering the full 17-STR profile.

**Figure 3:**
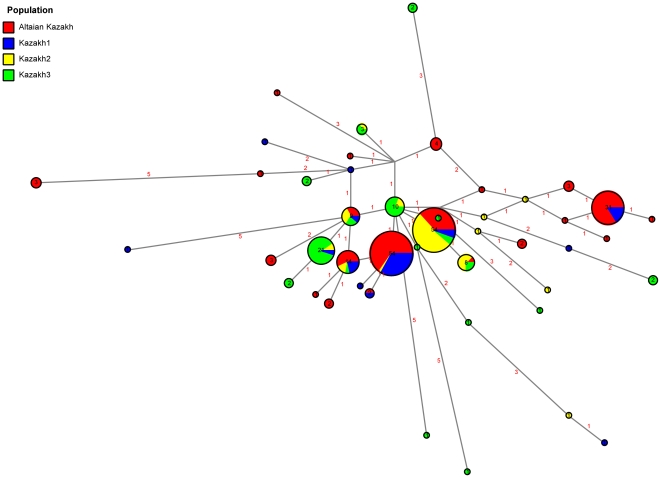
Reduced median-median joining network of Altaian and Indigenous Kazakh populations using 5-STR haplotypes.

We also reduced the 17-STR profile to a 5-STR profile (DYS389I, DYS390, DYS391, DYS392 and DYS393) to compare the Altaian Kazakh data with published data sets (**Figure 3**). As a result of this reduction, the 51 Altaian Kazakh haplotypes were collapsed into 21 haplotypes, and the number of shared haplotypes increased accordingly for haplogroups C3*, C3c and O3a3c*. Even so, R_ST_ values showed that SW and SE populations of Altaian Kazakhs remained distinctive even with a reduced number of Y-STRs under analysis (R_ST_ = 0.091; p-value = 0.005).

### Kazakh Haplotype Diversity

Once the Y-STRs were reduced to five locus profiles, we were able to compare our Altaian Kazakh population to those from the published literature [11], [12], [13]. Many of the Kazakh populations were characterized by only a few haplotypes. Of the 45 unique Y-STR haplotypes identified among all Kazakh populations, just five of them accounted for two-thirds of all of the Y-chromosomes in these groups. This fact explains the relatively low haplotype diversity estimated for these populations (**Table 3**). Interestingly, only two of these five haplotypes were found in all Kazakh populations, although they represented 27% of the entire Kazakh male gene pool. Based on the data sets that had biallelic marker and STR data (Altaian Kazakhs and Kazakh1), these two haplotypes belonged to haplogroup C3*, with the most frequent haplotype falling into the Genghis Khan haplotype cluster [14]. The remainder of the shared haplotypes belonged to haplogroups C3*, C3c and O3a3c*.

**Table 3:** Summary statistics among four Kazakh populations.

Population	Altaian Kazakh	Kazakh1	Kazakh2	Kazakh3
**N**	119	38	49	50
**Haplotypes**	21	13	13	17
**Haplotype Diversity**	0.835±0.020	0.760±0.068	0.665±0.074	0.844±0.043
**Pairwise Differences**	2.775±1.479	2.336±1.304	1.204±0.782	1.891±1.098
**References**	This study	[12]	[11]	[10]

The R_ST_ estimates provided evidence that the Kazakh populations are structured (**Table S2**). SE Altaian Kazakhs and SW Altaian Kazakhs were not significantly different from Kazakh1. They were also separated from Kazakh2 and Kazakh3 by large genetic distances (F_ST_ >0.139). This finding suggested that at least two subpopulations of Kazakhs exist in the regions of western Kazakhstan and the Altai Republic. Whether there are additional Kazakh subpopulations from central or eastern Kazakhstan remains to be determined.

### Coalescence Dating of NRY Haplogroups

To gain a better understanding of Kazakh Y-chromosome lineages, we analyzed haplogroups C3*, C3c and O3a3c*. The assessment of variation within these haplogroups was undertaken to better discern their origins, or the time at which they entered the Kazakh gene pool. While caution must be exercised when considering coalescence estimates calculated from Y-STR analysis [15], such estimates do provide relative values that are useful for making comparisons between populations or haplogroups. Thus, coalescence estimates were calculated using the rho statistic as implemented in Network 4.5.1.6 and through Bayesian analysis of a coalescent-based model with Batwing [16], [17].

The resulting estimates showed similarities between haplogroups and populations (Altaian Kazakh compared to indigenous Kazakh). Rho statistic estimates using the pedigree Y-STR mutation rate yielded coalescence dates for haplogroups C3* and C3c that were consistent with a source roughly 800 years ago (**Table 4**). Haplogroup O3a3c* had a much more recent TMRCA of approximately 400 years ago. These results were consistent for haplotypes from Altaian Kazakhs and indigenous Kazakhs, suggesting that these populations arose from a common source and experienced similar population histories. Furthermore, the comparable estimates for C3* and C3c and their occurrence in all Kazakh populations imply that both haplogroups were present in the ancestral population. Yet, the standard deviations for these estimates were large, and encompassed 800 to 1,300 years, depending on the data set. This time frame is centered close to the dates of the expansion of the Mongol empire [18], which also reflect the estimates generated by Zerjal et al. [14], [18]. By contrast, haplogroup O3a3c* appeared to represent a later expansion that would have occurred around the emergence of the Kazakh ethnic group.

**Table 4:** TMRCA estimates from 5-STR haplotypes using Rho statistics and Batwing.

Population	Hg	N	Network	Batwing – TMRCA		Batwing – Expansion	
**Pedigree-Based Mutation Rate**							
Altaian Kazakh	C3*	24	760±470	640	[270–1390]	430	[90–1260]
Kazakh4	C3*	40	780±550	1870	[590–5140]	1660	[260–5880]
Altaian Kazakh	C3c	47	830±630	1200	[480–2910]	1030	[170–3410]
Kazakh1	C3c	24	870±490	2350	[800–6560]	1750	[270–6020]
Kazakh5	C3c	14	370±370	450	[60–1760]	420	[30–2130]
Altaian Kazakh	O3a3c	31	420±280	410	[110–1200]	380	[50–1450]
**Evolutionary-Based Mutation Rate**							
Altaian Kazakh	C3*	24	2110±1320	1880	[730–4850]	480	[60–1910]
Kazakh4	C3*	40	2170±1520	6190	[1750–22,070]	3960	[670–13,870]
Altaian Kazakh	C3c	47	2310±1740	3630	[1280–10,830]	2300	[500–7430]
Kazakh1	C3c	24	2420±1350	6900	[2070–24,180]	3860	[700–13,640]
Kazakh5	C3c	14	1040±1040	1400	[170–6700]	1030	[90–5120]
Altaian Kazakh	O3a3c	31	1170±780	1550	[370–5500]	1070	[160–3960]

*TMRCAs were estimated using the rho statistic in Network v 4.5.1.6. TMRCA estimates using Batwing are represented by median values and 95% confidence intervals. All TMRCAs are expressed in years before present (BP).

Using the evolutionary mutation rate, we obtained coalescence estimates that were three times older than those calculated from the pedigree rate. Accordingly, the TMRCAs for haplogroups C3* and C3c were each over 2,100 years ago. At that time (when Greek and Roman historians were first recording the activities of nomadic steppe peoples), the Scythians and Sarmatians controlled much of Central Asia [3], [18]. Given the ancestral homelands of these later tribes in the West, it is likely that they would have brought NRY lineages with them to Central Asia during their expansions into the region. At the same time, Altaic speakers (Xiongnu) were moving westward [3], and could have brought with them C-derived Y-chromosomes.

In this regard, the TMRCAs based on rho statistics are likely to be underestimates. Median networks must assume that all haplotypes are identical by descent when, in fact, because of the high mutation rate of Y-STRs, there is good reason to believe that at least some could be identical by state. The Bayesian analysis generally gave higher estimates than calculations based on the rho statistic. The use of the pedigree mutation rates provided estimates between 600 and 2,300 years ago for haplogroups C3* and C3c, with expansion times 200 years later. Estimates for O3a3c* using the two methods were generally consistent. Such estimates have very broad 95% confidence intervals and, thus, cannot be used to precisely pinpoint the original source of these lineages.

The 14-locus profile used to generate extended haplotypes provided greater resolution for the Altaian Kazakhs and, thus, allowed additional analysis of its populations. For each of the three major haplogroups, haplotype clusters were identified using median network analysis. These clusters could represent clans within the Altaian Kazakhs similar to the “identity cores” described in Central Asian populations [11]. The TMRCA estimates based on 14-STR profiles were slightly younger than the results obtained from the 5-STR profiles, but were largely consistent with them (**Table 5**).

**Table 5:** TMRCA estimates of Altaian Kazakh haplotype clusters from 14-STR haplotypes using Rho statistics and Batwing.

Hg	N	Network	Batwing - TMRCA		Batwing - Expansion		
**Pedigree-Based Mutation Rate**							
C3*	20	470±120	260	[110–590]		280	[50–990]
C3c	35	480±240	400	[160–910]		420	[70–1440]
O3a3c	28	330±120	190	[60–490]		230	[30–880]
**Evolutionary-Based Mutation Rate**							
C3*	20	1290±590	930	[360–2330]		740	[140–2390]
C3c	35	1330±650	1130	[420–2860]		930	[160–2980]
O3a3c	28	920±320	810	[240–2270]		520	[80–1800]

*TMRCAs were estimated using the rho statistic in Network v 4.5.1.6. TMRCA estimates using Batwing are represented by median values and 95% confidence intervals. All TMRCAs are expressed in years before present (BP).

### Central Asian NRY Diversity

**Figure 4:**
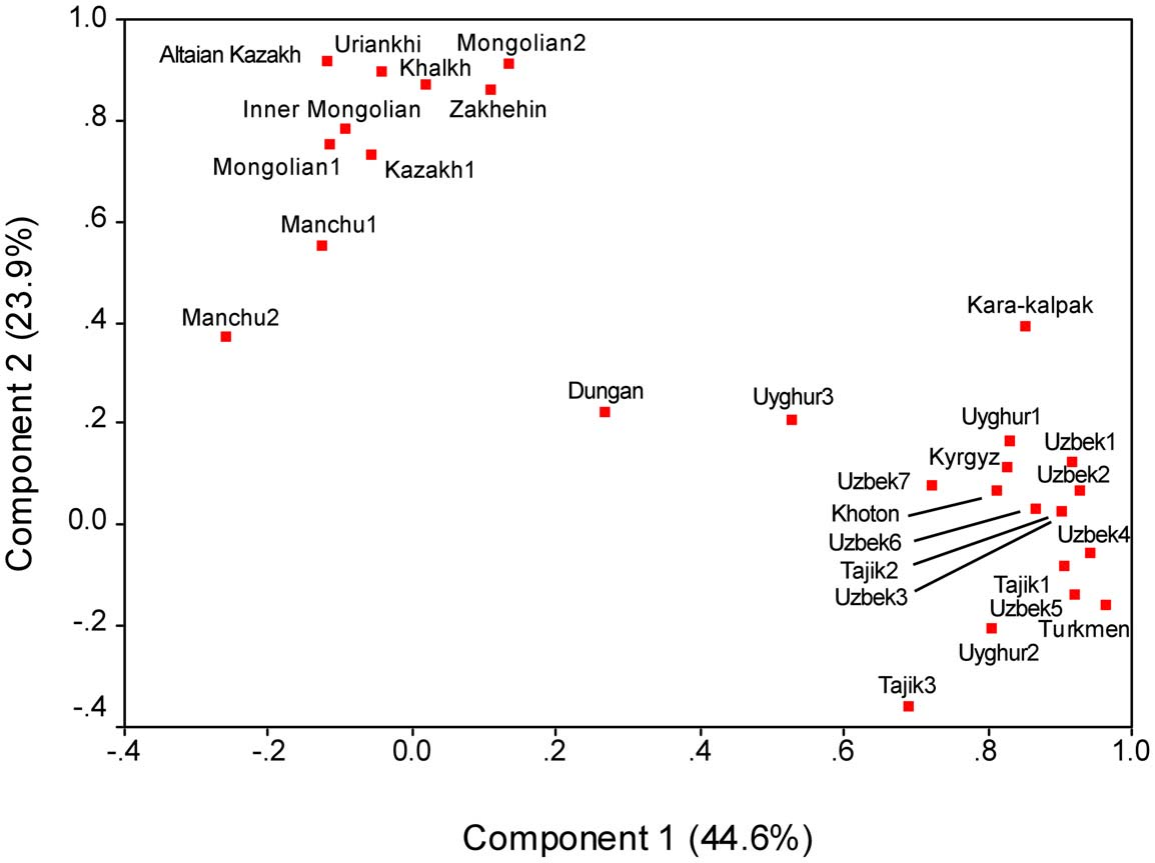
Principal component analysis plot of genetic distances based on Y-chromosome haplogroup frequencies in Central Asian and Mongolian populations.

Molecular diversity estimates and genetic distances were calculated to quantify the levels of genetic variation within and between Altaian Kazakhs and Central Asian populations. We used haplogroup frequencies from 28 populations for the principal components analysis (PCA) (**Figure 4**). Because the level of resolution differed across published studies, the data were reduced to 14-haplogroup profiles (see Methods). In the resulting PCA plot, the first component explained 44.6% of the variation, and grouped the Altaian Kazakhs with indigenous Kazakh and Mongolian ethnic groups at some distance from the remaining Central Asian populations. The second component explained 23.9% of the variation, and separated the Manchu from the Mongolian/Kazakh cluster, although it essentially reinforced the clusters of the first component.

**Figure 5:**
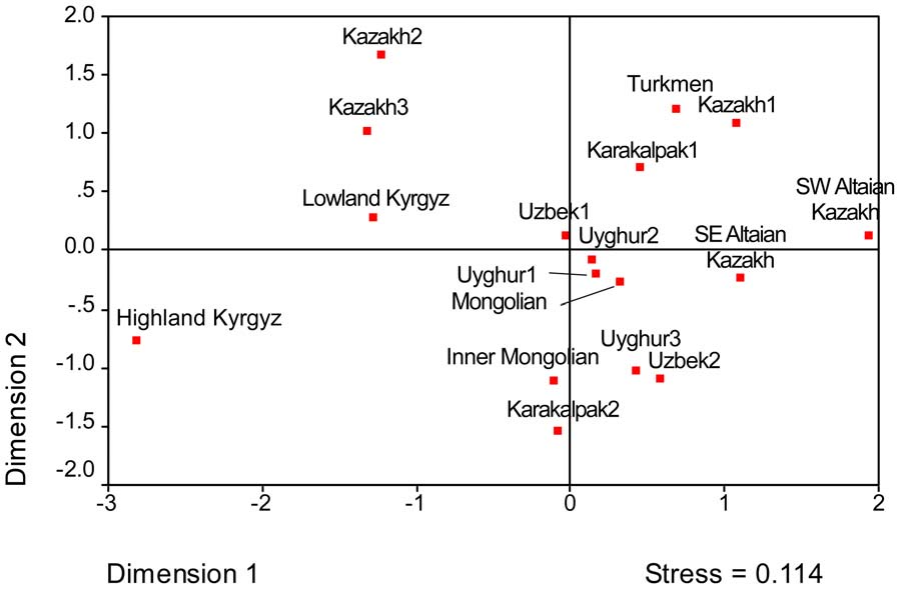
Multidimensional scaling plot of R_ST_ values estimated from Y-STR haplotypes in Central Asian and Mongolian populations.

Populations were also compared using R_ST_ estimates computed from the Y-STR data and plotted using multidimensional scaling (MDS) (**Figure 5**). The Y-STR data were taken from only five loci in order to include as many groups as possible from the published literature. R_ST_ estimates indicated that the Altaian Kazakhs shared the smallest genetic distance to the composite Kazakh1 and Uyghurs from Xinjiang, followed by Mongolians. Notably, Altaian Kazakhs (both from SW and SE) had limited genetic affinities with Central Asian populations aside from the Kazakh1. The remaining Kazakhs were positioned on the opposite side of the plot. Unlike the PCA, the Altaian Kazakhs were clearly separated from the indigenous Kazakh populations, with the latter showing greatest affinities with lowland Kyrgyz.

Comparisons of Central Asian Y-STR haplotypes provided additional evidence for the distinctiveness of the Kazakh Y-chromosome gene pool. The most frequent Altaian Kazakh haplotypes were shared with one or more of the three indigenous Kazakh populations. All but one of these haplotypes was also shared with Mongolians. Aside from the modal haplotypes, few were shared among Kazakhs and Central Asians (Kyrgyz, Uzbek, Kara-kalpak, and Turkmen). The Kyrgyz showed even greater differentiation from Central Asians than did Kazakhs. Thus, the Kazakhs and Kyrgyz are unique for Central Asia in not sharing many haplotypes with their neighbors. However, unlike the Kyrgyz, the populations with the greatest affinities to Altaian Kazakh populations were Mongolians.

Haplotype diversity and average pairwise differences indicated a lower level of genetic diversity among the Kazakh populations when compared to published data from other Central Asians. Turkmen and Kara-kalpak showed similarly low levels, with only the highland Kyrgyz being significantly less. These same populations showed the greatest distances from the central cluster in the MDS plot. All of these low-level diversity groups recently shared a similar semi-nomadic lifestyle, and are organized by patrilineal descent groups.

AMOVA was used to examine the partitioning of the genetic variation for these Central Asian and Mongolian populations (**Table 6**). Of the three categories analyzed (geography, language and ethnicity), ethnicity was the only category to produce significant values. In this case, 89.0% of the variation was found within groups, while about 3.6% of the variation was found in the “among group” category and 7.4% in the “among population within group” category. Variation in these partitions changed when the Kazakh populations were split into two groups (first group – SW Altaian Kazakhs, SE Altaian Kazakhs and Kazakh1; the second group – Kazakh2 and Kazakh3), based on the R_ST_ findings. This split resulted in an increase to 7.1% “among group” and a decrease to 4.1% for “among population within group.” About 12.8% of the variation was explained in the “among population within group” for the language and geography categories, with non-significant values for the “among group” category. The influence of geography on genetic variation was also explored using SAMOVA, but no clear geographic groupings showed significant “among group” values (data not shown).

**Table 6:** Analysis of molecular variance results of Y-STR haplotypes in Central Asian and Mongolian populations.

Groups	Percentage of Variation	P-value
**Geography**		
*Among group*	-0.29	0.412±0.002
*Between population within group*	13.29	0
*Within Group*	87.00	0
**Language**		
*Among group*	-2.61	0.908±0.001
*Between population within group*	12.87	0
*Within Group*	89.73	0
**Ethnicity**		
*Among group*	3.59	0.064±0.001
*Between population within group*	7.10	0
*Within Group*	89.31	0
**Ethnicity (Modified)**		
*Among group*	7.12	0.002±0.0001
*Between population within group*	3.61	0
*Within Group*	89.27	0

Note: Categories for “Geography” – Central Asia; Altai; Mongolia.

“Language” – Turkic; Mongolic.

“Ethnicity” – Kazakh; Kyrgyz; Uzbek; Uyghur; Kara-kalpak; Turkmen; Mongolian.

“Modified Ethnicity” – Altaian Kazakh + Kazakh1; Kazakh2 + Kazakh3; Kyrgyz; Uzbek; Uyghur; Kara-kalpak; Turkmen; Mongolian.

## Discussion

In this study, we characterized NRY variation in Altaian Kazakhs of the Altai-Sayan region of Siberia using high-resolution biallelic marker and STR analysis. To our knowledge, this is the highest NRY resolution data set for any Central Asian or Siberian population that has been published. The primary objectives of this study were to assess the paternal genetic variation in Altaian Kazakh populations and their population histories, to understand the paternal origins of Kazakhs, and to elucidate the process by which this ethnic group formed.

Altaian Kazakhs do not represent a single genetically isolated population. The paternal lineages present in the Altaian Kazakhs are generally distinctive from those appearing in indigenous populations among which the Kazakhs live. However, the interactions between indigenous Altaian and Kazakh groups differ between the two regions examined in this study. This difference, in turn, affects the overall amounts of genetic diversity within each Kazakh group and the extent of genetic relatedness they have with each other and their neighbors.

Interestingly, SW Altaian Kazakhs share NRY lineages with indigenous Altaians, while SE Altaian Kazakhs do not. While one must be cautious in interpreting these results due to the relatively small sample size of SW Altaian Kazakhs, this population still possessed higher levels of Y-chromosome diversity than the numerically larger SE Altaian Kazakhs. In addition, lineages not typically found in other Kazakh populations were present in the SW population. These findings were further supported by genealogical information collected in the field.

The samples from the SW region came primarily from one location. Historical evidence shows that this location is a community that consisted of Russian, indigenous Altaian and Kazakh individuals who have lived amongst each other for the past 150 years [5]. The conversion to Christianity of many of the Kazakhs in this community removed certain religious barriers to intermarriage. In addition, several waves of immigration by the Kazakhs over this time frame brought members of some 20 descent groups from the Middle *Zhüz* to this village [5]. Thus, this population appears to be a conglomeration of numerous Kazakh families. The multiple migrations into the southwest Altai region, along with the willingness of Kazakh groups to interact with non-Kazakh inhabitants in a diverse environment, help to explain the greater diversity within it. By contrast, language, culture and religion seem to have played a larger role in maintaining the separation of SE Altaian Kazakhs from their neighbors. Therefore, the differences observed in the Altaian Kazakhs are directly related to the manner in which they arrived in the Altai-Sayan region and the nature and extent of their interactions with the local indigenous populations.

Despite the differences observed among Altaian Kazakhs from these two regions of the Altai Republic, similarities at the haplogroup level were observed. Comparisons of Altaian Kazakhs and indigenous Kazakhs ultimately reveal a shared biological history. High frequencies of haplogroups C3* and C3c accompanied by the near absence of R1a1a* not only connect these Kazakh populations, but also sets them apart from other Central Asian populations. The ubiquity of haplogroup C-derived lineages in all Kazakh populations indicates that this ethnic group likely arose from a common source, even though political necessity – not common ancestry – defined the Kazakh Khanate in its beginning. Undoubtedly, not all of the lineages making up the Kazakh Khanate in its initial construction survived to the present, but at least a large portion of those that did survive are related.

Within this framework of common paternal ancestry, the Y-STR data highlight the fact that Kazakh populations are not entirely homogenous. Overall, the genetic differences between the two groups of Altaian Kazakhs are relatively small. However, they do share affinities with one set of indigenous Kazakhs (Kazakh1). Zerjal et al. [13] had previously noted close similarities between these Kazakhs sampled from several locations, but also commented on differences between their data set and that of Pérez-Lezaun et al. (Kazakh2) [12], [13]. Our observations reaffirmed these differences. Genetic distances showed that the first group (SE Altaian Kazakhs, SW Altaian Kazakhs and Kazakh1) has a greater affinity to Mongolians and Uyghurs, while the second group (Kazakh2 and Kazakh3) has a greater affinity to lowland Kyrgyz.

To some extent, these findings are not surprising. Differentiation of NRY haplotypes among populations (even within an ethnic group) is known to be common for pastoral Central Asian populations [19]. However, this fact does not explain the clustering of Kazakh populations into two groups. These differences hint at a more complex population history for Kazakhs.

One potential source for this distribution may have to do with the role that political organization (Kazakh *Zhüz*) has played in maintaining and/or redistributing genetic variation among Kazakh populations. These *Zhüz* were not based on common descent, but neither was the Kazakh Khanate in its infancy. In fact, the Kazakh Khanate reportedly formed from people associated with the Uzbek Khanate, which itself was an amalgam of Turkicized Iranian peoples, Eastern Kipchak nomads and Chagatai Turks [3], [20]. However, they currently lack any significant Y-chromosome affinities with Uzbeks (their putative source population).

Today, there is no doubt that Kazakhs share a common paternal source. It is possible that the observed differences were mostly maintained by these *Zhüz* affiliations, although they may simply result from sampling effects. In this regard, Kazakh1 represents a more heterogeneous collection of samples than the other indigenous Kazakh sample sets, having been obtained from four locations in southern and eastern Kazakhstan. Thus, this set of samples may more accurately represent the overall genetic diversity in Kazakhs. In other words, it may possibly include descendants from the Great and Middle *Zhüz*, whereas the others may only have descendants of the Great *Zhüz*. Therefore, comprehensive sampling of Kazakh populations throughout all of Kazakhstan and Xinjiang, China, is necessary to answer these questions conclusively.

Given the heterogeneous nature of the Kazakh Khanate at its inception, there are several explanations for how modern-day Kazakhs came to share a common paternal ancestry. First, the men that created the Kazakh Khanate could have possessed these lineages at a disproportionate frequency by chance, i.e. through a founder effect. Alternatively, the abundance of these lineages could be the result of a bottleneck that occurred during the beginning of the Khanate or soon thereafter. A third possibility is that some men could have had greater reproductive success, either through natural or “social” selection. Indeed, these scenarios need not be exclusive from one another.

The *süök* system, as employed by Kazakhs and other Turkic-speaking groups, provide a plausible explanation for the current patterns of Kazakh genetic diversity. Those who belong to the privileged *süök* were more likely to successfully retain larger herds, and thus be able to sustain larger families. Men of the privileged (or aristocratic) *süök* also claimed descent from Genghis Khan. NRY lineages (C3*) that putatively belong to Genghis Khan and his descendants are, in fact, found in high frequencies among the Kazakhs. Thus, these lineages could have spread by social selection [14], [21], [22]. While this interpretation is not definitive, we believe it is the most likely scenario.

Indeed, social structure has played an important role in shaping genetic variation in Central Asian populations. The cultural customs of descent lines, marriage and residence patterns significantly affect patterns of maternal and paternal lineage diversity [22]. These cultural elements also have the ability to affect the effective population sizes of a group [11], [22]. In this regard, pastoralist economies support fewer people at lower densities than do agricultural communities. Pastoralist communities that follow patrilineal descent and patrilocal residence patterns also retain low levels of paternal diversity [11], [12].

The small population sizes, in addition to the sex-specific nature of gene flow, produced the differences noted between the paternal and maternal genetic systems. As previously mentioned, only a small number of NRY haplogroups and haplotypes are present in Altaian Kazakhs, reflecting low levels of Y-chromosome diversity. However, the mtDNA evidence suggests that Altaian Kazakhs have a heterogeneous maternal ancestry, as would be expected given the patrilocal and patrilineal social structure of Kazakhs. Therefore, while the Kazakhs' social structure promoted change through genetic drift, social selection caused particular lineages (members of the privileged *süök*) to increase in frequency.

The mtDNA data further indicate that Altaian Kazakhs emerged from a common gene pool, which they share with other Central Asian and Mongolian populations [7], [23]. This gene pool was shown to be extremely diverse, with a variety of West and East Eurasian maternal lineages being present, further exemplifying the region's complex history. By contrast, the Y-chromosome data showed that Kazakhs and Mongolians diverged significantly from other Central Asian groups. Thus, the mtDNA and Y-chromosome genetic profiles indicate different, sex-specific contributions to Kazakh populations.

More generally, this study has added to our understanding of Altaic-speaking populations in Central Asia. It also allows us to address debates about the way in which Turkic languages spread in the region. Some believe that Turkic languages were spread from Eastern Eurasia westward, mostly by cultural diffusion in the form of elite dominance [10], [24], [25], [26], while others believe that both cultural and population replacement occurred [27]. Studies of mtDNA diversity provide evidence for an elite dominance pattern [8], [28], particularly for Anatolian populations [29], [30], [31]. The low frequencies of specifically “Central Asian” Y-chromosomes in Turkey add further support to this hypothesis [10], [26], [31].

The exact effect that Turkic language expansion(s) had on Central Asian populations is harder to untangle. NRY haplogroups C3* and C3c are found in nearly every Altaic-speaking population [10], [13], [32], [33], [34]. While one haplotype cluster is associated with Genghis Khan and his descendants, it is not possible to attribute the presence of all C-derived haplogroups in Central Asia to the actions of the Mongols. For example, ancient DNA studies have placed NRY haplogroup C in southern Siberia at roughly 1800 – 1400 BCE and from a Xiongnu cemetery in Mongolia at 100 BCE – 100 CE [35], [36]. The presence of this haplogroup in historical populations, both inside and outside of Mongolia prior to the 13^th^ century CE, indicate that it was present in the region prior to the emergence of the Mongol Empire, and thus, is not a signature of Genghis Khan's expansions. In fact, Zerjal et al. only attribute this haplotype cluster to Genghis Khan, not all haplogroup C Y-chromosomes [13].

Haplogroups R1 and Q are also well attested in Altaic-speakers [10], [13], [32], [33], [34]. While it is not clear whether Indo-Iranian speaking populations introduced R1 into Central Asia [37], this haplogroup appears at higher frequencies in Central Asia and southern Siberia than in Mongolia [10], [13], [32], [38]. Repeated migrations of Indo-Iranian, Turkic and Mongolic speakers into Central Asia in the form of Scythians, Sarmatians, Xiongnu, Turks, Mongols, and others provided new lineages and redistributed indigenous ones, such that historical Central Asian populations now represent an amalgamation of Y-chromosomes [10], [13]. The Kazakhs are unique in this genetic context in that their Y-chromosomes belong largely to the C3*, C3c and O3 haplogroups, and that these haplogroups were likely contributed by Altaic peoples moving westward from their homeland, presumably in southern Siberia or Mongolia. This lack of admixture (i.e., little to no P-derived haplogroups) contrasts dramatically with their heterogeneous mtDNA variation.

The difficulty in reliably determining the coalescent dates for the lineages found in Kazakh populations makes it nearly impossible to determine whether these lineages were present in ancestral nomadic steppe groups (Scythians, Xiongnu, Xianbei, Toba, and Jou-Jan) or were contributed by the descendents of Genghis Khan and the Mongol armies that, at one time, held control over the region. An important reason for caution here is the current debate about the most appropriate mutation rate for NRY coalescence estimates. The evidence provided by Zerjal et al. [14] supports the younger estimates, suggesting that the Kazakh haplotypes could be the direct result of the Mongol influence in the 13^th^ century CE. The presence of the C3* haplotype cluster in the Kazakh also supports the genealogical assertions that (for at least some Kazakh men) there is a direct paternal connection to Genghis Khan.

If the evolutionary rate is the more accurate value for Y-STRs, then the Kazakh lineages coalesce to roughly 2,000 years ago. This date suggests a far older source for them, possibly with the westward movements of Altaic-speaking peoples around the second and first centuries BCE. In this case, we would expect to see multiple haplotype clusters exhibiting a similar pattern as the Genghis Khan cluster. However, we do not observe this pattern. As Zerjal et al. [14] pointed out, this haplotype cluster is unique. Therefore, given the evidence presented here and in Zerjal et al. [14], we believe the best interpretation of the data is that Kazakh Y-chromosome diversity was strongly influenced by the Mongols of the 13^th^ century CE.

Ultimately, the genetic variation in Central Asian populations clearly depends on the social and cultural contexts within which these populations exist(ed). Populations that have small effective population sizes and follow patrilocal customs and patrilineal descent are prone to genetic drift while, at the same time, able to maintain a dominant patrilineal group composed of closely related men. Thus, while founder effects likely occurred during the ethnogenesis of the Kazakhs, their cultural practices subsequently shaped and maintained their paternal genetic diversity.

## Materials and Methods

Blood samples were collected from participants during several field expeditions conducted between 1991 and 2002. A total of 119 male Altaian Kazakh samples were collected from four locations in two regions of the Altai Republic, Russia (**Figure 1**). Genealogical relationships were recorded prior to sample collection, confirming that all participants were unrelated within at least the last two to three generations. All research was conducted with the approval of the University of Pennsylvania IRB and the Institute of Cytology and Genetics in Novosibirsk, Russia, with all samples being collected using informed consent written in Russian.

To elucidate the phylogeographic connections of Altaian Kazakhs to other populations in Central and East Asia, we compared their NRY data to those obtained from the published literature. For the haplogroup (biallelic marker) analysis, we used data from the following groups: Kazakh, Kyrgyz, Uzbek, Tajik, and Dungan [10], [13]; Kara-kalpak [10]; Inner Mongolian [39]; and Uyghur and Outer Mongolian [10], [13], [39]. For the haplotype (STR) analysis, we compared our data to those of the following groups: Kazakhs [11], [12], [13]; Kyrgyz [12]; Kara-kalpaks and Turkmen [11]; Uzbeks [11], [12]; Uyghur [12], [39]; and Outer and Inner Mongolian [39]. For the main comparisons between Altaian Kazakhs and indigenous Kazakh groups, i.e., those living in Kazakhstan, we designated those reported in Wells et al. [10] and Zerjal et al. [13] as “Kazakh1”, those from Pérez-Lezaun et al. [12] as “Kazakh2”, and those from Chaix et al. [11] as “Kazakh3.” For the coalescence analysis, C3* haplotypes for Kazakhs from Xinjiang, China, were obtained from Zhong et al. [40], and C3c haplotypes for Altaian Kazakhs were obtained from Malyarchuk et al. [41]. These were designated as “Kazakh4” and “Kazakh5”, respectively.

For comparisons with both biallelic marker and STR data, we condensed our high-resolution data set to make it compatible with those available in published studies. Thus, the paternal haplogroups were collapsed into 14 larger clusters that were more inclusive of the published data sets [C (xC3c), C3c, D, E, F (xJ), J, K (xN1c1, O, P), N1c1, O (xO1, O2a, O3) O1, O2a, O3, P (xRa1), and R1a]. Similarly, the STR haplotypes were reduced to six loci (DYS19, DYS389I, DYS390, DYS391, DYS392, and DYS393) to permit as broad a comparison as possible.

### Molecular Methods

Genomic DNA was extracted using standard phenol/chloroform methods [7]. The Y-chromosome of each participant was characterized using several methods. Most of the single nucleotide polymorphisms (SNPs) and fragment length polymorphisms were characterized using custom TaqMan assays (Applied Biosystems). These polymorphisms include (LLY22g, M3, M9, M12, M18, M20, M25, M45, M56, M69, M70, M73, M86, M89, M93, M102, M117, M119, M120, M122, M130, M134, M157, M170, M172, M173, M178, M201, M204, M207, M214, M242, M267, M269, M285, M217, M304, M323, M335, M346, M410, P15, P25, P31, P297 and PK2). Additional markers were detected through direct sequencing (M17, M343, M407, P39, P43, P48, P53.1, P62, P89, P98, P101 and PK5) and by PCR-RFLP analysis (M175) [42]. Seventeen short tandem repeats (STRs) were amplified using the multiplex AmpF*l*STR Yfiler PCR Amplification Kit (Applied Biosystems), and read on a 3130xl Genetic Analyzer with GeneMapper ID v3.2 software.

The assignment of each sample to NRY haplogroups followed the conventions outlined by the Y Chromosome Consortium [43], [44]. Here, the C3* designation was used for Y-chromosomes having the markers for C3 (M217 and PK2) but lacking markers for C3a, C3b, C3c, C3d, C3e and C3f (M93, P39, M86, M407, P53.1 and P62, respectively). Similarly, O3a3c* Y-chromosomes were those derived for M134, but which had the ancestral state for M117 and P101. Each paternal haplotype was designated by its 17-STR profile. In this context, we define “lineages” as the unique combinations of SNP and STR data. For all statistical and network analyses, we used data from DYS389b by subtracting DYS389I from DYS389II [45]. In addition, we excluded DYS19 from the statistical analysis because it is duplicated in some haplogroups, particularly haplogroup C3c.

### Statistical Analysis

Summary statistics were calculated using Arlequin v3.11 [46]. Gene diversity (or haplotype diversity) was estimated using STR data sets. Principal components analysis (PCA) was conducted with haplogroup frequencies to assess the genetic similarity of the comparative populations using SPSS 11.0.0 [47]. In addition, pairwise differences between haplotypes, R_ST_ values between populations and AMOVA were calculated using Arlequin v3.11. R_ST_ values were calculated from STR haplotypes and visualized using multidimensional scaling (MDS) with SPSS 11.0.0.

AMOVA was used to assess the amount of genetic variation partitioned “among groups”, “among populations within groups” and “within populations” for geographic, ethnic and linguistic categories. The nature of genetic variation within Altaian Kazakh and that between them and indigenous Kazakh populations was examined, with Altaian Kazakhs being placed in one group and the indigenous Kazakhs in the other. Geographic structuring of diversity was also explored using three regional groupings: (1) Altai region, including Altaian Kazakhs and indigenous Altaians; (2) Central Asia; and (3) Mongolia/Northern China. We also used two linguistic categories, with Central Asians, Kazakhs and Altaians belonging to one group (Turkic) and Mongolians to a second (Mongolic). Finally, we analyzed variation in populations based on their respective ethnic group membership.

### Coalescence Dating of NRY Haplogroups

Lineages consisting of biallelic markers and STR data provided the basis for the phylogenetic analysis. Relationships between haplotypes were studied using Network 4.5.1.6 and Network Publisher 1.2.0.0 [16]. The weighting scheme employed relied on the amount of variation per locus, i.e., the weight of each locus increased inversely to the variance of allele repeats at that locus for each network [48]. We used a combined reduced median-median joining technique for all networks, while also focusing on haplogroups C3*, C3c and O3a3c*. The relative extent of diversity within each haplogroup was assessed using two methods. The first involved rho statistics, as implemented in Network 4.5.1.6, and the second employed a coalescent-based Bayesian analysis in Batwing [17].

The time to the most recent common ancestor (TMRCA) and expansion times were calculated with Batwing. The prior distributions for Batwing follow those established in previous studies, and were run for 50,000 cycles, with the removal of a 5,000 cycle burn-in [39]. The convergence of posterior distributions was assessed by increasing the length of run times by 10x, since this allowed us to determine whether the posterior values we obtained had stabilized [49]. Haplogroup TMRCAs were calculated using 5-STR haplotypes with haplogroup membership confirmation by biallelic marker characterization. The 14-STR profiles were used to examine the TMRCAs of haplotype clusters within haplogroups C3*, C3c and O3a3c* for Altaian Kazakhs. Batwing runs used a model assuming the initial population maintained a constant size, then expanding at time β with a growth rate of α. TMRCAs for specific haplogroups were calculated using a scaled population size equal to the frequency of the haplogroup in the population [50]. Both the evolutionary and the pedigree-based mutation rates were used to estimate coalescence dates with generation times of 25 and 30 years, respectively [51], [52], [53].

## Supporting Information

Table S1 17 Y-STR haplotypes for Altaian Kazakhs.
(PDF)

Table S2 R_ST_ value matrix of Central Asian and Mongolian populations.
(PDF)
