# Supplementary material for: Y-Chromosome Variation in Altaian Kazakhs Reveals a Common Paternal Gene Pool for Kazakhs and the Influence of Mongolian Expansions
Source: PLoS One. 2011 Mar 11;6(3):e17548. doi: 10.1371/journal.pone.0017548 (PMC3055870; doi:10.1371/journal.pone.0017548)
Supplement: Table S1 — 17 Y-STR haplotypes for Altaian Kazakhs. (PDF) [file pone.0017548.s001.pdf]

Table S1: 17 Y-STR haplotypes for Altaian Kazakhs

Haplotype #	Haplogroup	DYS19	DYS385	DYS389I	DYS389II	DYS390	DYS391	DYS392	DYS393	DYS437	DYS438	DYS439	DYS448	DYS456	DYS458	DYS635	Y GATA H4	Southwestern	Southeastern
1	C3*	16	12-13	13	29	25	10	11	13	14	10	10	23	15	18	21	11	1	2
2	C3*	17	12-13	13	29	25	10	11	13	14	10	10	22	15	18	21	11	2	
3	C3*	16	12-13	13	29	25	10	11	13	14	10	10	22	15	18	21	11	6	1
4	C3*	16	12-13	13	29	25	10	11	13	14	10	10	22	15	17	21	11	1	
5	C3*	16	12-12	13	29	25	10	11	13	14	10	10	22	15	18	21	11		4
6	C3*	15	12-14	14	30	24	10	11	13	14	11	10	20	17	17	21	11		1
7	C3*	16	12-13	13	29	26	10	11	13	14	10	10	22	15	19	21	11		1
8	C3*	16	12-13	13	29	25	10	11	13	14	10	10	21	15	18	21	11		1
9	C3*	15	12-14	14	30	24	10	11	13	14	11	10	X	18	17	21	11		1
10	C3*	15	12-14	14	30	24	10	11	13	14	11	10	X	17	17	21	11		1
11	C3*	16	12-13	13	30	25	10	11	13	14	10	10	23	16	18	21	11		1
12	C3*	16	12-13	13	29	25	10	11	13	14	10	10	23	15	17	22	11		1
13	C3c	15-16	12-12	15	32	24	9	11	13	14	10	11	20	15	18	24	10	1	
14	C3c	15-16	12-12	13	30	24	9	11	13	14	10	12	20	15	19	24	10	2	
15	C3c	15-16	12-12	14	31	24	9	11	13	14	10	12	20	15	19	24	10	1	
16	C3c	16	12-12	14	31	23	9	11	13	14	10	11	20	15	19	23	10	1	
17	C3c	15-17	12-12	14	31	23	9	11	13	14	10	11	20	15	19	23	10	1	
18	C3c	15-16	12-12	13	30	24	9	11	13	14	10	11	20	15	19	24	10		1
19	C3c	15-17	12-12	13	30	24	9	11	13	14	10	11	20	15	19	24	10		18
20	C3c	15-17	12-12	13	30	24	9	11	13	14	10	11	20	15	18	24	10		6
21	C3c	16-17	12-12	14	31	24	9	11	13	14	10	11	20	15	18	24	10	1	
22	C3c	15-17	12-12	13	30	24	9	11	13	14	10	11	20	15	18	25	10		1
23	C3c	17-18	12-12	14	30	24	9	11	13	14	10	11	21	15	18	23	10		1
24	C3c	15	12-12	14	31	24	9	11	13	14	10	11	20	15	18	24	10		1
25	C3c	15-17	12-12	14	31	24	9	11	13	14	10	11	20	15	18	24	10		3
26	C3c	15-17	12-12	13	30	24	9	11	13	14	10	12	20	15	19	24	10		1

Haplotype #	Haplogroup	DYS19	DYS385	DYS389I	DYS389II	DYS390	DYS391	DYS392	DYS393	DYS437	DYS438	DYS439	DYS448	DYS456	DYS458	DYS635	Y GATA H4	Southwestern	Southeastern
27	C3c	15-17	12-12	13	30	24	9	11	13	14	10	11	20	15	19	25	10		3
28	C3c	15-17	12-12	13	30	24	9	11	13	14	10	12	20	15	18	23	10		1
29	C3c	15-17	12-12	13	30	24	9	11	13	14	10	11	X	15	19	24	10		1
30	C3c	15-17	12-12	14	31	24	9	11	13	14	10	11	20	15	20	24	10		1
31	C3c	15-17	12-12	13	29	25	9	11	13	14	10	11	20	15	19	24	10		1
32	C3c	15-17	12-12	13	31	24	9	11	13	14	10	11	20	15	19	24	10		1
33	G1	13	13-17	14	29	23	10	12	13	16	10	13	22	16	15	20	11	3	
34	G1	13	13-17	14	29	23	11	12	13	16	10	12	22	16	15	20	11		1
35	G2	15	16-16	13	29	22	10	10	13	16	10	11	21	16	17	21	11	2	
36	J2a	14	15-16	13	29	23	10	11	12	15	9	11	21	15	18	23	11	1	
37	J2a	14	13-16	13	29	24	11	11	12	15	9	11	19	15	20	21	11		4
38	O3a3c*	15	13-18	12	28	23	10	13	12	15	10	12	19	15	17	20	12	1	
39	O3a3c*	15	13-18	12	29	22	10	13	12	15	10	12	19	15	17	19	12		1
40	O3a3c*	15	13-18	12	29	23	10	13	12	15	10	12	19	15	17	19	12		19
41	O3a3c*	15	13-17	12	29	23	10	13	12	15	10	12	19	15	17	19	12		3
42	O3a3c*	13	12-15	12	28	23	10	12	12	15	11	11	19	15	17	19	11		3
43	O3a3c*	15	14-18	12	29	23	10	13	12	15	10	12	19	15	17	19	12		1
44	O3a3c*	15	13-18	12	29	23	10	13	12	15	10	13	19	15	17	19	12		1
45	O3a3c*	15	13-18	13	30	23	10	13	12	15	10	12	19	15	17	19	12		1
46	O3a3c*	15	13-18	12	29	23	10	13	12	15	10	12	19	15	17	20	12		1
47	Q1a3*	13	15-16	13	30	23	10	14	12	13	11	11	20	15	16	22	11	1	
48	R1a1*	16	11-14	13	29	23	11	11	13	14	11	10	20	15	15	23	12	1	
49	R1b1b1	14	13-13	14	30	19	11	13	13	14	10	13	19	15	17	24	10	2	
50	R1b1b1	14	13-13	14	30	19	11	13	13	15	10	13	19	15	17	24	11	1	
51	T	15	13-15	13	29	23	11	13	13	14	9	11	18	17	15	21	11	1	
