# Supplementary material for: Y-Chromosome Variation in Altaian Kazakhs Reveals a Common Paternal Gene Pool for Kazakhs and the Influence of Mongolian Expansions
Source: PLoS One. 2011 Mar 11;6(3):e17548. doi: 10.1371/journal.pone.0017548 (PMC3055870; doi:10.1371/journal.pone.0017548)
Supplement: Table S2 — RST value matrix of Central Asian and Mongolian populations. (PDF) [file pone.0017548.s002.pdf]

Table S2: R_{ST} value matrix of Central Asian and Mongolian populations.

	SW Alt Kazakh	SE Alt Kazakh	Kazakh2	Kazakh3	Kazakh1	Uzbek1	Uzbek2	Highland Kyrgyz	Lowland Kyrgyz	Uyghur1	Uyghur2	Uyghur3	Turkmen	Kara-kalpak1	Kara-kalpak2	Mongolian	Inner Mongolian
SW Altaian Kazakh	*	.005	.000	.000	.051	.073	.010	.000	.000	.271	.797	.118	.028	.072	.007	.089	.002
SE Altaian Kazakh	.091	*	.000	.000	.302	.004	.001	.000	.000	.117	.018	.000	.000	.000	.000	.001	.000
Kazakh2	.228	.252	*	.206	.000	.002	.000	.000	.032	.000	.000	.000	.000	.000	.000	.000	.000
Kazakh3	.160	.252	.010	*	.000	.010	.000	.001	.426	.000	.001	.000	.000	.000	.000	.000	.000
Kazakh1	.050	.049	.158	.139	*	.005	.001	.000	.000	.016	.090	.000	.006	.004	.000	.007	.000
Uzbek1	.047	.089	.102	.075	.088	*	.108	.000	.058	.653	.449	.126	.113	.089	.028	.135	.030
Uzbek2	.085	.103	.218	.192	.141	.029	*	.000	.000	.401	.212	.248	.000	.001	.311	.123	.409
Highland Kyrgyz	.300	.444	.243	.120	.387	.172	.253	*	.018	.000	.000	.000	.000	.000	.000	.000	.000
Lowland Kyrgyz	.136	.223	.036	-.001	.143	.036	.130	.053	*	.002	.006	.000	.000	.000	.000	.001	.001
Uyghur1	.008	.052	.138	.113	.059	-.012	-.001	.197	.079	*	.930	.564	.043	.097	.118	.707	.099
Uyghur2	-.020	.049	.128	.095	.029	-.002	.014	.191	.072	-.023	*	.525	.084	.188	.084	.873	.086
Uyghur3	.026	.113	.241	.186	.134	.023	.008	.234	.123	-.007	-.006	*	.000	.001	.208	.104	.028
Turkmen	.063	.114	.146	.115	.071	.024	.151	.343	.120	.038	.029	.126	*	.247	.000	.001	.000
Kara-kalpak1	.036	.117	.130	.112	.069	.030	.102	.252	.122	.024	.014	.090	.007	*	.000	.007	.000
Kara-kalpak2	.095	.191	.251	.205	.196	.060	.003	.212	.145	.020	.028	.010	.186	.122	*	.024	.103
Mongolian	.027	.079	.151	.121	.065	.019	.017	.233	.094	-.010	-.016	.019	.074	.052	.037	*	.106
Inner Mongolian	.108	.135	.174	.151	.130	.052	-.002	.217	.101	.024	.029	.045	.171	.124	.022	.018	*

R_{ST} values are located in the lower matrix. P-values were calculated from 100,000 permutations are located in the upper matrix. Comparative samples came from published sources: SW Altaian Kazakhs [this study], SE Altaians Kazakhs [this study], Kazakh1 [13], Kazakh2 [12], Kazakh3 [11], Uzbek1 [12], Uzbek2 [11], Highland Kyrgyz [11], Lowland Kyrgyz [11], Uyghur1 [12], Uyghur2 [39], Uyghur3 [39], Turkmen [11], Kara-kalpak1 [11], Kara-kalpak2 [11], Mongolian [39], Inner Mongolian [39].
